# The role of Whitnall’s ligament position in the success of levator resection surgery in congenital ptosis

**DOI:** 10.1186/s12886-023-03238-z

**Published:** 2023-12-05

**Authors:** Mansooreh Jamshidian Tehrani, Abolfazl Kasaee, Haniyeh Zeidabadinejad, Mansoor Shahriari, Seyed Mohsen Rafizadeh

**Affiliations:** 1 Ophthalmology, Eye Research Center, Farabi Eye Hospital, Tehran University of Medical Sciences, Tehran, Iran; 2 Ophthalmology, Imam Hossein Medical Center, Ophthalmic Research Center, Shahid Beheshti University of Medical Sciences, Tehran, Iran

**Keywords:** Ptosis, Levator muscle resection, Whitnall’s ligament, Levator function, Levator resection surgery

## Abstract

**Purpose:**

This study aimed to investigate the role of Whitnall’s ligament position in the success of levator resection surgery in congenital ptosis.

**Methods:**

It was an interventional case series on patients with congenital ptosis who underwent levator muscle resection in Farabi Eye Hospital (2020–2022). Patients with incomplete follow-up, a history of trauma, poor Bell’s phenomenon, previous ocular and lid surgeries, poor levator function (≤ 4mm), and syndromic ptosis or systemic diseases were excluded. During the surgery, several factors, including the distance between Whitnall’s ligament and the upper edge of the tarsus (W-distance), the vertical length of the tarsus (T-length), and the amount of levator muscle resection (LMR), were measured. A successful outcome was defined as the inter-eye difference of margin reflex distance-1 (MRD1) ≤ 1 and post-op MRD1 ≥ 3 OR the inter-eye difference of MRD1 ≤ 0.5 with any value of post-op MRD1 in unilateral cases and Postop-MRD1 > 3 in bilateral cases during the 3-months period.

**Results:**

Thirty four eyes of 34 patients were included, and 79.4% of patients achieved successful outcomes. In univariate analysis, Preop-MRD1 and Preop-LF had meaningful negative correlations with the amount of LMR to reach the successful outcome (*p* < 0.05), which was only meaningful for Preop-LF in multivariable analysis (*p* < 0.05). Noticeably, W-distance had a significant positive correlation in univariate and multivariable linear regression (*p* < 0.05).

**Conclusions:**

W-distance can be considered a significant new parameter other than Preop-LF influencing the amount of levator resection needed to achieve success in levator resection surgery.

## Introduction

Blepharoptosis as a malpositioned (lower than normal) upper eyelid is considered congenital if evident in the early years of life. It can be either unilateral or bilateral, isolated or associated with other ocular or systemic diseases [1].

Congenital ptosis, with a prevalence of about 0.18% to 1.41% [2, 3], is often idiopathic; however, familial and autosomal dominant inheritance patterns have been reported. It is unilateral in 75% of cases, and 20% result in amblyopia due to either the deprivation effect of the ptosis or induced astigmatism [4]. Surgical treatment is often delayed until the patient reaches preschool age. However, if the ptosis is severe or the clinical signs of early amblyopia are evident and cannot otherwise be treated early surgery is considered [5]. Choosing an appropriate surgical plan (frontalis suspension technique vs. levator muscle procedures) depends on multiple factors, including the function of the Levator, other lid or face abnormalities, and clinical features of the ptosis [6].

Levator resection is suitable for patients with preserved levator function [7]. The outcomes are not fully predictable. However, Multiple factors such as the age, severity of ptosis, and levator function have been discussed, and formulas have been proposed to predict surgery results [8–10]. There is still space for other variables that play a role in the success of the surgery. The importance of Whitnall’s ligament was discussed by Anderson et al. in 1979 as the check ligament of the levator and a supportive structure for the upper eyelid and superior orbit Functionally [11]. There are few studies to investigate the association between levator muscle and Whitnall’s ligament biometrics and surgical success rate and postoperative outcome [11]. In fact, it is important to investigate why performing levator resection above Whitnall's ligament in patients with similar levator muscle function can lead to different results and whether the position of Whitnall's ligament plays a role in this regard. This study aimed to investigate possible factors (such as Whitnall’s ligament anatomical position) that may play a role in the outcomes of levator resection surgery.

## Methods

This study was a prospective interventional case series on patients with congenital ptosis presenting to the oculoplastic department of Farabi Eye Hospital (May 2020-January 2022) who underwent levator muscle resection. The study was approved by the institutional review board of the Tehran University of Medical Sciences and adhered to the tenets of the Declaration of Helsinki. Written informed consent was taken from all participants. The inclusion criteria were simple congenital ptosis. Patients with incomplete follow-up, a history of trauma, poor Bell’s phenomenon, previous ocular and lid surgeries, poor levator function (≤ 4mm), and syndromic ptosis (Marcus Gunn Jaw Winking or Blepharophimosis syndrome) or systemic diseases were excluded. All of the included patients underwent history taking and complete ophthalmic examination, including visual acuity measurement, slit-lamp biomicroscopy, funduscopy, evaluation of ocular movements and bell’s phenomenon, measuring of margin reflex distance-1 (MRD1), and amount of levator function (LF) in millimeter. The amount of levator function (LF) was measured about 1 week before surgery in the oculoplastic clinic. For this purpose, while the patient was in a sitting position and the physician was carefully in front of the patient, the physician restrained the frontalis muscle action by pressing his hand on the forehead and above the eyebrow of the patient. Then, The LF was assessed by measuring the amount of eyelid excursion from extreme down to up-gaze (mm) by a ruler, while the zero number of the ruler was equivalent to the upper eyelid margin in down-gaze. The severity of ptosis was determined based on the MRD1 of the ptosis eye before surgery. Accordingly, if 2mm < MRD1, it was considered as mild ptosis, and if 1mm < MRD1 ≤ 2mm, it was considered as moderate ptosis, and if MRD1 ≤ 1mm, it was considered as severe ptosis.

Candidate patients underwent a levator resection procedure by two oculoplastic surgeons (M.J.T. and S.M.R.). During the surgery, several factors were measured and recorded, including the distance between Whitnall’s ligament and the upper edge of the tarsus (W-distance), the vertical length of the tarsus (T-length), and the amount of levator muscle resection (LMR). MRD1 measurements were repeated after surgery. Pre-operative and post-operative photographs (in the three-month follow-up) of patients were taken and recorded while they were looking straight across. MRD1 measurements were done by ImageJ software with a scale of the horizontal corneal diameter of 12 mm from recorded photographs or directly by a ruler at the office. Post-operative follow-up visits were scheduled for ten days,1 and 3 months after surgery.

### Surgical technique and measurement

First, the upper eyelid crease was marked with a marker, and then local anesthesia, including 1 to 2 cc of lidocaine mixed with adrenaline (with a concentration of 1 in 100,000), was injected. After putting a traction suture in the upper eyelid margin with the 4–0 silk, the skin was incised at the place that was marked, and dissection was performed under the orbicularis muscle. Then, the septum opened, and after the fat prolapse, the levator muscle was identified under it.

**Fig. 1:**
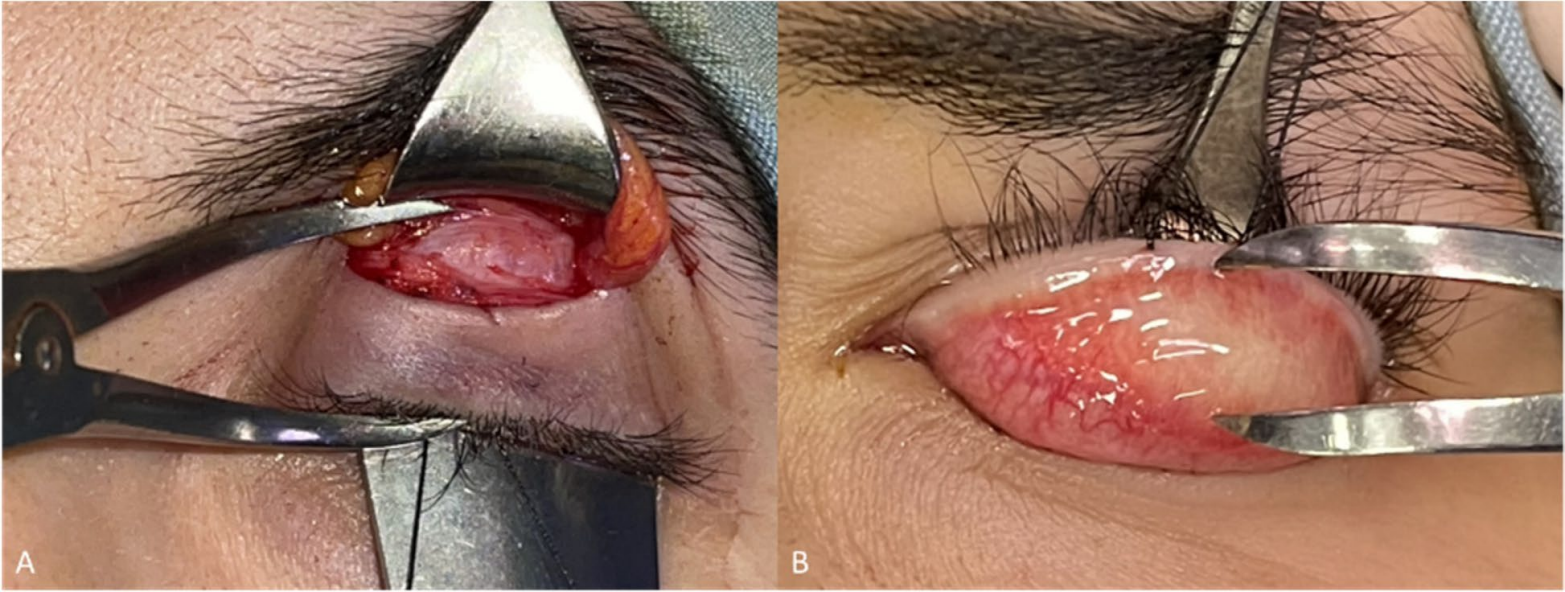
For Whitnall’s distance measurement, first the fat was pushed back by placing a desmarres and then using a caliper, the distance between Whitnall’s ligament and the upper lid margin was measured (**A**). Then, the eyelid was turned back, and the T-length was measured from the inside of the eyelid using a caliper (**B**). By subtracting the second distance from the first, W-distance was obtained

The dissection between the fat and the levator muscle continued up to a few millimeters above the location of Whitnall’s ligament. For measurement, first the fat was pushed back by placing a desmarres and then using a caliper, the distance between Whitnall’s ligament and the upper lid margin was measured. Then, the eyelid was turned back, and the T-length was measured from the inside of the eyelid using a caliper. By subtracting the second distance from the first, W-distance was obtained (Fig. 1A-1B). Then, the levator muscle was separated from the tarsus, and the continuation of the surgery was performed routinely. With three double arms vicryl 5–0 sutures, passing between the upper third and the lower two-thirds of the tarsus and then through the levator muscle, the levator was fixed from the new location to the tarsus. Adult patients were asked to sit, and the amount of resection was modified based on the surgeon's experience and according to the severity of ptosis and levator function. In children, the surgery was performed under general anesthesia, and the upper eyelid margin was adjusted approximately at the level of the upper limbus. The distance between the lower edge of the levator and the central suture was considered the amount of levator resection (LMR). The skin was repaired using 6–0 nylon sutures, and in order to form an eyelid crease, the remaining end of the levator muscle was taken in sutures. Then, the eye was bandaged with antibiotic eye ointment. The next day, the patient was visited, and lubricant drops and antibiotic eye ointment were prescribed. In addition, the patient was visited ten days, four weeks, and three months after the operation.

A successful outcome was defined as the inter-eye difference of MRD1 ≤ 1 and post-op MRD1 ≥ 3 OR the inter-eye difference of MRD1 ≤ 0.5 with any value of post-op MRD1 in unilateral cases and Postop-MRD1 > 3 in bilateral cases.

### Statistical analysis

We used frequency and percent, mean ± SD, and range to describe the data. We used correlation coefficient and simple linear regression to assess the univariate relation of different parameters on the amount of levator muscle needed to be resected to achieve success in levator muscle resection surgery. In addition, we used multivariable linear regression to evaluate the simultaneous (adjusted) effect of variables on this outcome. A P-value less than 0.05 is considered statistically significant. All statistical analysis was performed by SPSS (IBM SPSS Statistics for Windows, Version 23.0. Armonk, NY: IBM Corp.).

## Results

After the exclusion of 12 patients, 34 eyes of 34 patients were entered into the study analysis. The mean age of the patients at the surgery time was 25 ± 12 years (range 6–55 years). Four patients had bilateral ptosis in whom only one eye underwent ptosis surgery. Descriptive analysis, including intraoperative parameters such as Preop-LF, W-distance, T-length, LMR, Preop-MRD1, Postop-MRD1, MRD1-Change, and Inter-eye diff of MRD1, are summarized in Table 1. Based on the severity of ptosis 20.6% of patients had mild ptosis, 58.8% had moderate ptosis, and 20.6% had severe ptosis. 27 out of 34 patients (79.4%) achieved successful outcomes.

**Table 1:** Description of all parameters

Parameter	Mean ± SD (mm)	Range (mm)	
		Min	Max
Preop-LF	9.2 ± 3.3	4.5	18
W-distance	15.2 ± 3.4	8	23
T-length	7.5 ± 1.0	5	10
LMR (Levator Muscle Resection)	13.4 ± 4.6	7	24
Preop-MRD1	1.6 ± 0.7	0	3.3
Postop-MRD1	3.2 ± 1.2	0.0	6
MRD1-Change	1.5 ± 1.2	0.0	4.5
Inter-eye diff of MRD1	0.7 ± 0.6	0	2.9

**Fig. 2:**
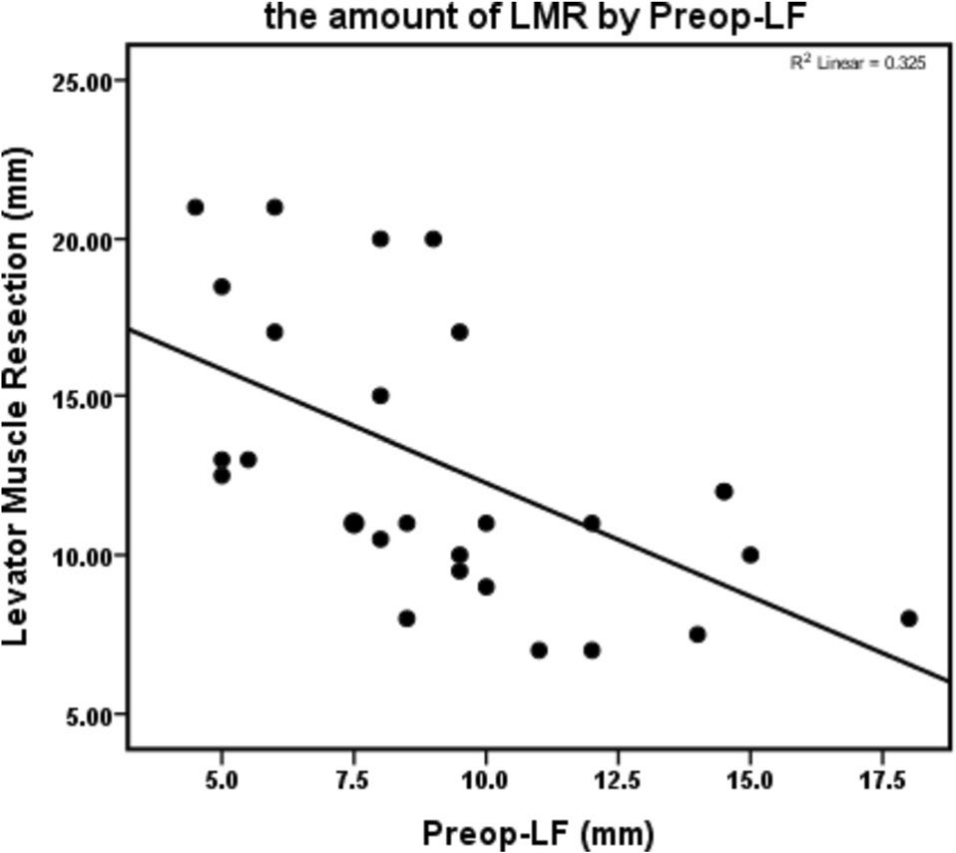
Scatterplot showing pre-operative levator function (Preop-LF) and the corresponding amount of levator muscle resection (LMR), r.^2^ = 0.325, *p* < 0.05

**Fig. 3:**
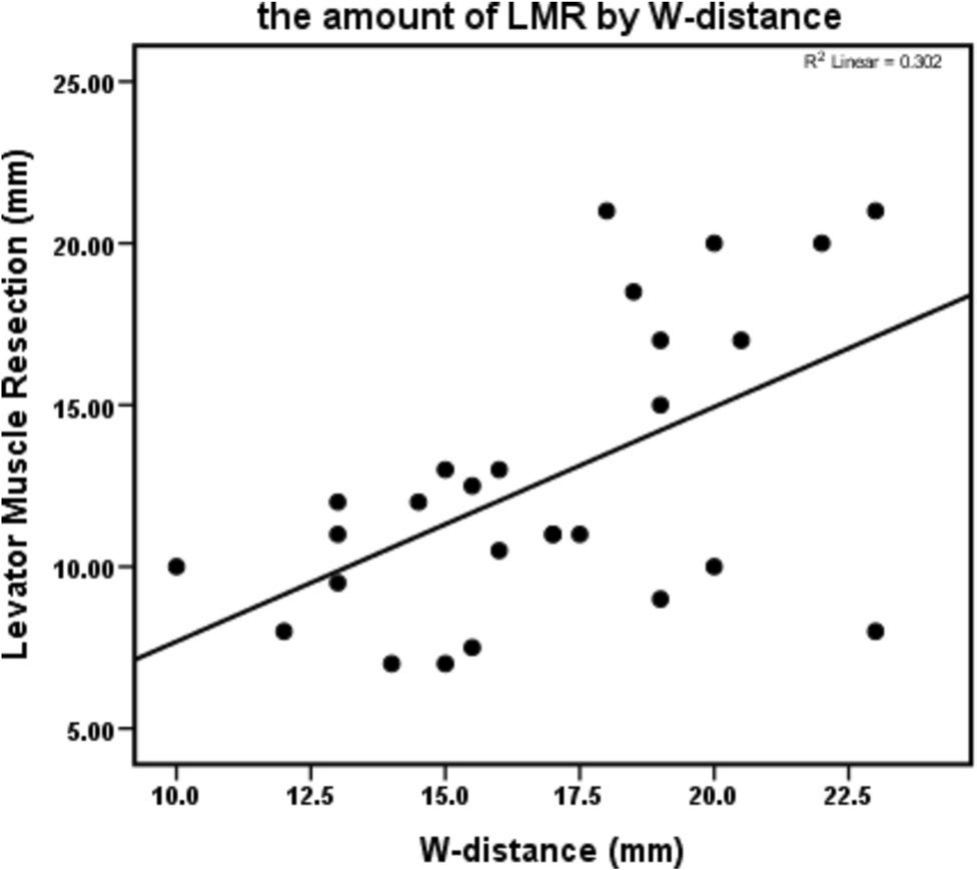
Scatterplot showing Whitnall” s ligament distance (W-distance) and the corresponding amount of levator muscle resection (LMR), r.^2^ = 0.302, *p* < 0.05

On the issue of correlation between different parameters and the amount of LMR to reach the successful outcome in the levator muscle resection surgery (*n* = 27 patients), T-length showed no statistically meaningful correlation (*P* > 0.05). In univariate analysis, Preop-MRD1 and Preop-LF had meaningful negative correlations, which was only meaningful for Preop-LF in multivariable analysis (*p* < 0.05). Noticeably, W-distance had a significant positive correlation in both univariate and multivariable linear regression (Table 2) (Figs. 2 and 3).

**Table 2:** Univariate and adjusted (simultaneous) effect of different parameters on the amount of levator muscle resection (Beta: Regression Coefficient)

Parameter	Correlation		Simple linear regression				Multivariable linear regression			
			Beta	95% CI		P	Beta	95% CI		P
	r	P		Lower	Upper			Lower	Upper	
Preop-MRD1	-0.395	0.041	-2.547	-4.98	-0.10	0.041	-0.970	-3.195	1.255	0.376
Preop-LF	-0.570	0.002	-0.713	-1.137	-0.290	0.002	-0.490	-0.932	-0.049	0.031
W-distance	0.549	0.003	0.725	0.271	1.179	0.003	0.496	0.015	0.976	0.044
T-length	0.014	0.943	0.064	-1.762	1.890	0.943	-0.199	-1.682	1.284	0.783

## Discussion

Under correction or over-correction is a considerable point in ptosis surgery. A large amount of levator resection may result in a shortening of the distance between the tarsus and the Whitnall’s ligament as the check ligament of the levator palpebral superioris muscle leading to eyelid lag and lagophthalmos [12].

The amount of levator resection depends on various factors such as pre-operative levator function and ptosis severity. However, accurate prediction of outcome is still difficult [13, 14], and Although a variety of formulas have been introduced to determine the amount of levator resection, there is no consensus on these formulas due to unpredictability [8, 15].

During surgery, Whitnall’s Ligament is characterized as a landmark at the most superior extent of the aponeurosis. It is located in a region almost between the muscular part of the levator muscle and its aponeurotic part. This transverse fibrous white band lied from the medial to the lateral border of the levator aponeurosis immediately behind the superior orbital rim. In some cases of ptosis correction, this structure may be utilized to improve levator resection surgery efficiency and even used as a sling to support the eyelid at a higher level. Anatomic variability in this structure may contribute to ptosis [16]. Also, these variabilities of levator aponeurosis and Whitnall’s ligament can influence surgical outcomes. The distance between the insertion of the levator muscle and Whitnall’s ligament has been reported to be 14–20 mm in previous studies [17]. However, standard values for Asians are not available.

According to the findings of our study, Preop-LF had a significant negative correlation with the amount of levator muscle resection. It was consistent with the results of Berke 1961 [9], Beard 1966 [8], and Also, the study of Cates and Tyers, which was conducted on congenital ptosis in children and adults and showed that the best predictor of the results of levator resection surgery is the pre-operative levator function [13]. The study by Nguyen and colleagues in 2017 on patients with congenital ptosis showed ptotic eyes with lower Preop-LF needed a higher amount of levator muscle resection [10]. They found there was a correlation between pre-operative levator function and the amount of levator resection; however, this correlation was not strong. Based on this, they stated that there are probably factors other than levator function that influence the amount of levator resection required to achieve a successful outcome in levator resection surgery [10]. According to the results obtained from our study, one of these factors can be Whitnall’s distance. In fact, in our study, we noticed a positive correlation between Whitnall’s distance and the amount of levator resection, so by increasing the distance between Whitnall’s ligament and the upper edge of the tarsus, the amount of levator resection required during surgery for successful outcome, increases. Perhaps this finding can be explained considering the constant length of the levator muscle by increasing the distance between Whitnall’s ligament and the tarsus, the percentage of the levator aponeurosis increases and the percentage of its muscular part decreases, so the overall strength of the muscle decreases, and therefore, a greater amount of resection is needed to correct ptosis.

Another hypothesis proposed by the authors is that this positive correlation can be explained using the lever rule. The comparison of body muscle movements to levers in humans and animals has been investigated in previous studies [18, 19]. However, no studies have discussed it in ophthalmology. In fact, the posterior part of the levator is muscular and extends horizontally from its origin to Whitnall’s ligament. The anterior part of the levator, which consists of the aponeurosis, extends vertically from Whitnall’s ligament to the insertion site. In addition, Whitnall’s ligament, which has connections to the roof and sides of the orbit, acts as a support that converts the horizontal force of the levator muscle into a vertical force [11]. The posterior (muscular) part of the levator can be compared to the effort arm of the lever, and the anterior (aponeurotic) part of the levator can be compared to the resistant arm of the lever. Accordingly, at a fixed length of the lever, by increasing the resistance arm (the distance of Whitnall’s ligament from the upper edge of the tarsus) and decreasing the effort arm, it is necessary to resect a greater amount of the levator so that the ratio of the effort arm length to the resistance arm length increases further, leading to being more effective in correcting ptosis.

Based on our results and clinical experience, W-distance can be considered an influencing factor in the surgical outcome during levator resection surgery. Patients with lower values of Preop-LF and higher values of W-distance may benefit from higher amounts of levator muscle resection (*p* < 0.05). Based on Figs. 2 and 3, as well as the values obtained for correlation analysis, it seems that Whitnall's distance has an almost equal and opposite effect to the effect of levator function on the amount of resection required to achieve success in levator resection surgery. To the best of our knowledge, there are few studies on the evaluation of the effect of W-distance on the amount of levator muscle resection. One may hypothesize that patients with higher values of W-distance may have a poorer LF. However, our study did not show a correlation between these two parameters, and W-distance may affect the resection amount in ways other than LF. In our study, Preop-MRD1 and T-length were not effective on the amount of levator resection.

Overall, according to Beard, success in levator resection surgery depends on several factors [8, 20]. The most important factor in this success is the perfect decision of the surgeon during surgery. In fact, the surgeon determines the amount of levator muscle resection based on the type of ptosis, the severity of ptosis, and the amount of levator muscle function. Primarily, the experience of the surgeon plays a very important role, and the number of sutures and the placement of the sutures are very important in creating an acceptable final contour for the upper eyelid. So far, no study has mentioned the decisive role of Whitnall’s ligament in the success of levator resection surgery. Therefore the anatomical position of Whitnall’s ligament, which was mentioned in our study, should not replace the role of proven variables, Like levator muscle function or surgeon experience. In fact, our study is just the beginning of a way and points to the possible but not definitive role of Whitnall’s ligament in levator resection surgery. There is definitely a need for further and more detailed studies with a larger number of cases to check the existence or absence of this relationship.

Remarkable aspects of our study were the novel idea and prospective design. The limitations of this study were the relatively small sample size. Future studies with larger sample sizes and randomization are required to show the different aspects of this topic better. Nowadays, novel automatic image processing techniques in eyelid and face parameter measurements, for example, 3D models, algorithmic innovation, multi-classification models, and deep learning methods, have made great progress and can contribute to the level of diagnosis and decision-making and help us a lot in the field of diagnosis and treatment of eyelid and face diseases [21–24].

The results of this and future studies can be considered by oculoplastic surgeons to estimate better and faster the needed amount of levator muscle resection to achieve more successful outcomes in levator resection surgery.

In conclusion, this study showed the W-distance as a significant new parameter influencing the amount of levator resection needed to achieve success in levator resection surgery. Patients with higher W-distance or weaker Preop-LF may benefit from higher amounts of levator resection.

## Data Availability

The datasets used and/or analyzed during the current study are available from the corresponding author upon reasonable request.
